# Demographic differences among patients treated with chimeric antigen receptor T‐cell therapy in the United States

**DOI:** 10.1002/cam4.4797

**Published:** 2022-05-08

**Authors:** Josephine Emole, Odunayo Lawal, Oleksandra Lupak, Ajoy Dias, Leyla Shune, Korede Yusuf

**Affiliations:** ^1^ Stem Cell Transplantation and Cellular Therapy Henry Ford Hospital Detroit Michigan USA; ^2^ Clinical and Translational Science Program University of Arizona Tucson Arizona USA; ^3^ Hematology/Oncology University of South Carolina Charleston South Carolina USA; ^4^ Blood and Marrow Transplantation Beth Israel Deaconess Hospital Boston Massachusetts USA; ^5^ Hematological Malignancies and Cellular Therapeutics The University of Kansas Health System Kansas City USA; ^6^ Public Health Program Adelphi University Garden City New York USA

**Keywords:** CART cell therapy, disparities, leukemia, lymphoma, multiple myeloma, sociodemographic factors

## Abstract

**Background:**

It is not clear if all Americans have benefitted equally from the availability of chimeric antigen receptor T‐cell (CART) therapy. We aimed to evaluate if demographic differences existed among adult patients who received CART therapy and to assess predictors of CART treatment outcomes.

**Methods:**

Records of patients ≥18 years who received CART therapy for non‐Hodgkin’s lymphoma, acute lymphoblastic leukemia, and multiple myeloma in 2018 were evaluated in the National Inpatient Sample. Acute complications and inhospital mortality were compared between two groups of CART recipients: Whites and non‐Whites. Logistic regression analysis was used to evaluate the association between sociodemographic factors and inhospital mortality.

**Results:**

Of 1275 CART recipients that met inclusion criteria, there were 40.4% of females, 66.9% of Whites, Blacks (4.2%), Hispanics (13.3%), Asians or Pacific Islanders (4.2%), and Native Americans (1.3%). Up to 96.8% of CART procedures were performed in urban teaching hospitals, and 85.3% of CART recipients lived in metropolitan counties. Non‐Whites, compared to Whites, were younger at the time of CART therapy (*p* < 0.001). The inhospital mortality rate was higher in non‐Whites, though not statistically significant (5.4% vs. 4.4%, *p* = 0.764). There were no differences in length of hospital stay, hospital charges, or rates of acute toxicities between the two race groups. We found no association between race and treatment outcomes. Gender, neurotoxicity, and Charlson Comorbidity Index were significant predictors of inhospital mortality.

**Conclusions:**

CART therapy recipients in the United States were more likely to be Whites and more likely to be residents of metropolitan areas. These observed demographic differences were not associated with treatment outcomes or inhospital mortalities.

## Graphical abstract

The first CART product was approved for commercial use in 2017, but it is not clear if all Americans have benefitted equally from the availability of CART therapies. In this retrospective study, we evaluated the demographic characteristics of CART therapy recipients for the year 2018. We found that racial minorities (especially Blacks) and people who live outside metropolitan areas were less likely to receive CART therapies.

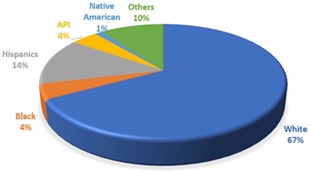

## 1 INTRODUCTION

Chimeric antigen receptor T‐cell (CART) therapy is an adoptive cellular therapy used for the treatment of chemotherapy‐refractory hematological malignancies. Most of the CART therapies currently in use are produced from autologous T cells obtained from patients by apheresis, genetically modified to target a specific tumor antigen, expanded, cryopreserved, and eventually infused back into patients. Prior to infusion of CART cells, patients may receive lymphodepleting chemotherapy. While cytokine release syndrome and neurotoxicity are common toxicities following CART infusion^1^, other longer term complications include cytopenia and B‐cell aplasia with hypogammaglobulinemia. CART therapy is associated with considerable financial costs, irrespective of the site of administration—inpatient or ambulatory settings.^2^, ^3^, ^4^

The availability of CD19‐directed CART has led to impressive response rates for patients with relapsed/refractory B lymphomas. In the ZUMA‐1 trial, which evaluated the efficacy of axicabtagene ciloleucel in 111 patients with refractory diffuse large B‐cell lymphoma (DLBCL), primary mediastinal B‐cell lymphoma, or transformed follicular lymphoma, the investigators reported an objective response rate of 82% (complete response rate of 54%). With a median follow‐up of 15.4 months, a 40% complete response rate was observed.^5^ Tisagenlecleucel was evaluated in a multicenter phase 2 study for adult patients with relapsed or refractory DLBCL who were ineligible for or had disease progression after autologous hematopoietic stem cell transplantation. After a median follow‐up of 14 months, an overall response rate of 52% was observed (40% had complete responses, while 12% had partial responses).^6^ Another CART product (lisocabtagene maraleucel) was evaluated in the TRANSCEND trial for adult patients with relapsed or refractory large B‐cell lymphoma (DLBCL, double‐hit or triple‐hit lymphoma, DLBCL transformed from any indolent lymphoma, primary mediastinal B‐cell lymphoma, or follicular lymphoma grade 3B). The authors reported an objective response rate of 73%, with 53% of patients achieving a complete response.^7^

In addition to currently approved indications in high‐grade B‐cell lymphomas, CART therapies have been approved for B‐cell acute lymphoblastic leukemia, mantle cell lymphoma, follicular lymphoma, and multiple myeloma.^8^, ^9^, ^10^, ^11^, ^12^ While there are currently no approved CART products for other hematological disorders, these therapies are being evaluated for CLL,^13^, ^14^, ^15^ Hodgkin's lymphoma^16^, ^17^, ^18^, and AML.^19^, ^20^, ^21^ Beyond autologous CART therapies, allogeneic CART cells obtained from healthy donors and genetically edited to target respective antigens are actively being evaluated.^22^

Despite the remarkable responses observed in CART therapy for hematological malignancies, these outcomes may not be experienced equally by all Americans. In the United States (US), it is known that certain populations suffer health disparities more than others.^23^ Minority populations (Asians, American Indians, Alaskan Natives, Hispanics, and Blacks) have been known to experience poorer health compared to their Caucasian counterparts.^24^, ^25^ Persons without health insurance have a lower likelihood of having an usual source of health care, preventative care, and management of chronic conditions,^26^ while patients who are uninsured or underinsured at the time of diagnosis of cancer are more likely to die in 5 years compared to patients with private insurance.^26^ For hematological malignancies, racial and socioeconomic factors have been found to affect access to and outcomes of treatment.^27^

Very few studies have evaluated the effect of sociodemographic characteristics on the receipt and outcomes of CART therapy for adult patients with hematological disorders. Initial data from the Center for International Bone Marrow Transplant Research (CIBMTR) on CART utilization show that minority groups were underrepresented among recipients of CART therapies for hematological disorders.^28^ A pediatric real‐world consortium data showed that African American children were less likely to undergo CART therapy (even after apheresis) and had poorer outcomes after CART therapy.^29^ Since these studies were not specific to the adult CART population, it is not clear how much their conclusions could be extrapolated to adult recipients. To effectively evaluate CART receipt among adults in the US and inform interventions that will address any gaps and disparities, dedicated studies of CART utilization patterns and outcomes have become necessary.

Our study aimed to evaluate if demographic differences existed among patients that received CART therapy for non‐Hodgkin lymphoma, multiple myeloma, and acute lymphoblastic leukemia—three disease groups with the most frequent CART use in recent years.^28^ This study also sought to compare CART treatment outcomes between racial groups and evaluate the association of sociodemographic characteristics and inhospital mortality.

## 2 METHODS

### 2.1 Data source

For this study, we used data from the National Inpatient Sample (NIS). The NIS is the largest publicly available all‐payer inpatient care database in the US and provides regional and national estimates of inpatient utilization, charges, quality, and outcomes. As a stratified sample, the NIS contains 20% (over 7 million) of all discharges from nonfederal acute care hospitals for the year 2018. When weighted, it estimates more than 35 million hospitalizations in the US. The 2018 NIS sampling frame includes data from 48 statewide data organizations (47 states plus the District of Columbia).^30^ Each discharge record in the NIS contains patient‐level data (demographics, diagnoses, procedures, insurance, discharge, total hospital charges, and length of hospital stay) as well as hospital‐level data (geographic region, location, teaching status, bed size, and ownership). All data in the database are de‐identified and compliant with the Health Insurance Portability and Accountability Act.

### 2.2 Ethics approval statement

Prior to commencing this study, ethical approval was sought from the Institutional Review Board of Henry Ford Hospital, Detroit, Michigan, and the study received an exemption determination.

### 2.3 Study design

From the NIS database, we selected and included all hospitalization records for patients 18 years or older, who underwent CART therapy for acute lymphoblastic leukemia (ALL), multiple myeloma (MM), or non‐Hodgkin's lymphoma (NHL) from January 2018 to December 2018. The year 2018 was selected for analysis, being the first full year after CART became available for commercial use. Hospitalization records for patients who received CART infusions without a diagnosis of MM, NHL, or ALL were excluded from the analysis. The International Classification of Disease, Tenth Revision (ICD‐10) procedure codes for autologous CART immunotherapy were used to identify CART procedures. ALL, MM, and subtypes of NHL were identified using their respective ICD‐10 diagnostic codes. Our study was limited to autologous CART products, and the study design did not make a distinction between first and subsequent CART procedures. Additionally, our study did not differentiate between commercially available and investigational CART products.

Patient‐level data extracted from eligible hospital records were age, gender, race, hematological disorders, insurance, income, location of patients’ residence, and patient comorbidities. Hospital level characteristics of interest were hospital geographical location and teaching status. Charlson Comorbidity Index was used as a summary measure of patients’ comorbidities. Income was represented by the median household income quartiles for the patient’s zip code. The location of patients' residences was assessed by the National Center for Health Statistics (NCHS) urban–rural classification scheme for counties in the United States. Counties in areas with a population ≥50,000 were considered metropolitan (urban). For metropolitan areas of ≥1 million population, the “fringe” counties were designated as suburban counties. Counties in areas of <50,000 population were designated nonmetropolitan (rural).

The primary study outcome was CART utilization patterns across the US. Secondary outcomes were inhospital mortality and rates of acute CART treatment complications (hemophagocytic lymphohistiocytosis, neurotoxicity, sepsis, acute kidney injury, fever, and respiratory failure). Assignment of neurotoxicity was guided by the consensus grading for neurological toxicity by the American Society for Transplantation and Cellular Therapy.^1^ Sociodemographic factors, comorbidities, CART treatment complications, and hospitalization outcomes were compared between two groups of CART recipients: Whites and non‐Whites (Black, Hispanic, Asian, Pacific Islander, Native American, and others).

### 2.4 Statistics

Clinical variables were summarized using counts and proportions for categorical data and median and interquartile ranges for continuous data. In compliance with the NIS data use agreement, categories were combined, and some data were suppressed to avoid reporting any cell counts less than or equal to 10. Since the NIS is a stratified sample, appropriate weights were applied to the analyses to obtain national estimates. All variables were checked for missing data, and frequencies of missing data were reported. Chi‐squared (χ^2^) test was used to compare categorical variables, while continuous variables were compared between race groups (White vs. non‐White) using the Wilcoxon rank‐sum test. Prior to statistical modeling, the distribution of missing data was reported, and the multiple imputation method^31^ was used to impute missing data, resulting in a database with no missing data. Race had the highest level of missingness (5.5%). Regression methods were used to evaluate the association between patient sociodemographic factors and inhospital mortality, and odds ratio (OR) with 95% confidence intervals (CI) was reported. Potential confounders such as location of residence, insurance, age, gender, and comorbidity index were controlled for. All tests were two sided, and the results were considered significant at an alpha level of 5% (*p* < 0.05). Stata version 16 (*StataCorp*) was used for statistical analysis.

## 3 RESULTS

### 3.1 Baseline patient and treatment characteristics

Of the 1425 autologous CART infusions identified in the database for the year 2018, 1275 met the inclusion criteria for our study. The analytic population (N = 1275) consisted of adults aged 18–82 years, of whom 515 (40.4%) were female. The sociodemographic and clinical characteristics of the CART recipients are stratified by cancer diagnosis; acute lymphoblastic leukemia (ALL)[n = 115], multiple myeloma (MM)[n = 200], and non‐Hodgkin's lymphoma (NHL)[n = 960] are presented in Table 1. The median age of patients was 59 years with an interquartile range of 18 years. Patients older than 70 years comprised 10.2% of the study population. CART recipients included Whites (66.9%), Blacks (4.2%), Hispanics (13.3%), Asians or Pacific Islanders (4.2%), Native Americans (1.3%), and others (10%). Non‐Hodgkin's lymphoma (diffuse large B‐cell lymphoma) was the most common indication for CART treatment in the year 2018. Primary payer was private insurance for 752 (59%) patients, Medicare for 337(26.4%), and Medicaid for 129(10.1%) patients. With regard to acute CART toxicities, neurotoxicity was reported in 395 (30.9%) patients, sepsis in 130 (10.2%), acute kidney injury in 180 (14.1%), fever in 630 (49.4%), respiratory failure requiring mechanical ventilation in 80 (6.3%), and hemophagocytic lymphohistiocytosis in 15 (1.2%) patients.

**TABLE 1:** Demographic and clinical characteristics of hospitalized CART recipients (NHL, ALL, and MM) in 2018

Characteristics *n* (%)	NHL	ALL	MM	All patients
Total	960 (75.3)	115 (9.0)	200 (15.7)	1275 (100.0)
Age in years, median (IQR)^a^	60 (14.5)	23 (6)	59 (10)	59 (18)
Gender				
Female (%)	385 (40.1)	60 (52.2)	70 (35.0)	515 (40.4)
Race				
Whites	666 (69.4)	46 (40)	142 (71.1)	853 (66.9)
Blacks	21 (2.2)	S	32 (15.8)	54 (4.2)
Hispanics	99 (10.4)	52 (45)	21 (10.5)	169 (13.3)
Asian or Pacific Islander	53 (5.5)	S	S	54 (4.2)
Native American	11 (1.1)	S	S	17 (1.3)
Others	110 (11.5)	S	S	128 (10.0)
Location of patient's residence				
Central counties of metro areas of ≥1 million	270 (28.1)	36 (31.8)	92 (46.2)	395 (30.9)
Fringe counties of metro areas of ≥1 million population	228 (23.8)	42 (36.4)	72 (35.9)	343 (26.9)
Counties in metro areas of 250,000–999,999 population	213 (22.2)	16 (13.6)	21 (10.3)	250 (19.6)
Counties in metro areas of 50,000–249,999 population	83 (8.7)	S	S	100 (7.8)
Nonmetropolitan (<50,000 population)	166 (17.3)	S	S	187 (14.7)
Median household income				
$1–$45,999	146 (15.2)	42 (36.4)	15 (7.7)	204 (16.0)
$46,000–$58,999	225 (23.4)	31 (27.3)	36 (18.0)	292 (22.9)
$59,000–$78,999	324 (33.7)	26 (22.7)	67 (33.3)	418 (32.7)
$79,000 or more	265 (27.7)	16 (13.6)	82 (41.0)	361 (28.3)
Charlson Comorbidity Index				
1–2	545 (56.8)	65 (56.5)	140 (70.0)	748 (58.7)
≥3	415 (43.2)	50 (43.5)	60 (30.0)	527 (41.3)
Hospital Location (US region)				
Northeast	290 (30.2)	15 (13.1)	90 (45.0)	396 (31.1)
Midwest	205 (21.4)	15 (13.0)	15 (7.5)	236 (18.5)
South	265 (27.6)	30 (26.1)	35 (17.5)	332 (26.0)
West	200 (20.8)	55 (47.8)	60 (30.0)	311 (24.4)
Hospital type				
Urban teaching hospital	935 (97.4)	115 (100)	190 (95)	1234 (96.8)

Abbreviations: NHL, Non‐Hodgkin's lymphoma; ALL, acute lymphoblastic leukemia; MM, multiple myeloma; HMO, health maintenance organization; US, United States; S, suppressed data, IQR, Interquartile Range.

^a^ Median (IQR) reported.

### 3.2 Comparison of clinical variables between racial groups

Due to the low number of patients in some racial/ethnic groups and in compliance with the data use agreement, this study combined all minority racial/ethnic groups, so that comparisons were focused on two groups: Whites and non‐White (Black, Hispanics, Pacific Islanders, Asians, Native Americans, and others). In Table 2, we present the comparison of select variables between CART recipients, by race. Non‐Whites, compared to Whites, were younger at the time of CART therapy (median age 56 years vs. 61 years, *p* < 0.001) and had higher Charlson Comorbidity Index score (49.5% vs. 36.7%, *P* = 0.021). Whites were more likely to live in nonmetropolitan counties compared to non‐Whites (17.1% vs. 10.4%, *p* = 0.040). While the proportion of patients with private insurance was similar between the two groups, Whites were twice more likely to have Medicare (33.1% vs. 15.4%, *p* < 0.001). Inpatient mortality was 4.7% (60 patients) and was comparable for both groups. The median length of hospital stay for the entire cohort was 15 days (range 3–112 days) and did not differ between the two groups (15 days for Whites and 16 days for non‐Whites, *p* = 0.204). Gender, median total hospital charges, and rates of acute complications were not significantly different for Non‐Whites compared to Whites. The rate of discharge disposition to short‐term hospitals, skilled nursing facilities, or intermediate care facilities was 6.8% for Whites and 3.2% for non‐Whites, *p* = 0.124.

**TABLE 2:** Comparison of select variables between CART recipients in 2018

Variable	White (%)	Non‐white (%)	*p*
Age in years median (IQR)^a^	61 (13)	56 (24)	< 0.001
Location of patient's residence			0.04
Central counties of metro areas of > = 1 million population	23.42	44.83	
Fringe counties of metro areas of > = 1 million population	31.65	18.39	
Counties in metro areas of 250,000–999,999 population	20.25	18.39	
Counties in metro areas of 50,000–249,999 population	07.59	08.05	
Nonmetropolitan (<50,000 population)	17.09	10.35	
Female	37.27	46.24	0.254
Charlson Comorbidity Index			0.021
0–2	63.35	50.54	
≥3	36.65	49.46	
Inhospital mortality	04.35	05.38	0.737
Discharge to other facilities^b^	06.83	03.23	0.124
Acute CART treatment complications			
Hemophagocytic lymphohistiocytosis	01.18	01.86	0.140
Neurotoxicity	30.43	32.26	0.729
Sepsis	11.8	07.53	0.224
Acute kidney injury	12.42	17.2	0.308
Fever	49.69	49.46	0.978
Respiratory failure	06.21	06.45	0.935
Length of stay in days (median, days)	15	16	0.204
Total charges (median, US dollars)	537,281	493,887	0.521

IQR, Interquartile Range.

^a^ Median (IQR) reported.

^b^ Short‐term hospital, skilled nursing facility, intermediate care facilities.

### 3.3 Bivariable and multivariable analyses

In Table 3, we present the results of select predictors of inhospital mortality (unadjusted and adjusted odds ratios). In the unadjusted model, the significant predictors of inhospital mortality were neurotoxicity and Charlson Comorbidity index ≥3. Race, insurance type, income, and gender did not show any significant association with inhospital mortality. After adjusting for potential confounders, gender, neurotoxicity, and Charlson Comorbidity index ≥3 were predictors of inhospital mortality. Compared to patients with Charlson Comorbidity of 0–2, those with an index ≥3 had about five times higher odds of mortality (OR = 5.09, 95% CI: 1.71–15.13, *p* = 0.004). Similarly, the presence of neurotoxicity was associated with higher odds of mortality (OR = 14.05, 95% CI: 4.20–46.96, *p* < 0.001), while females had a 72% lower likelihood of death compared to males (OR = 0.28, 95% CI: 0.12–0.68, *p* = 0.005). No association was found between sociodemographic factors and acute toxicities, length of hospital stay, or total hospital charges.

**TABLE 3:** Select predictors for inhospital mortality

Variable	Unadjusted OR	CI	Adjusted OR	CI
Female (versus Male)	0.47	0.16–1.41	0.28	0.12–0.68
White (versus non‐white)	0.80	0.22–2.95	0.68	0.12–3.96
CCI ≥3(versus CCI 0–2)	4.24	1.69–12.32	5.09	1.71–15.13
Location of patient's residence				
Central countries of metro areas of ≥1 million population	Ref		Ref	
Fringe counties of metro areas of ≥1 million population	1.56	0.52–4.79	1.30	0.31–5.55
Counties in metro areas of 250,000–999,999 population	1.06	0.23–4.79	0.40	0.06–2.69
Counties in metro areas of 50,000–249,999 population	1.35	0.14–13.50	0.42	0.06–2.89
Non‐metropolitan areas (<50,000 population)	1.43	0.11–6.77	0.31	0.03–3.13
Neurotoxicity (versus no neurotoxicity)	7.37	1.99–27.27	14.05	4.20–46.96

OR, odds ratio; CI, confidence interval; CCI, Charlson Comorbidity Index; Ref, reference category.

## 4 DISCUSSION

Our study represents one of the first and largest inquiries into the patterns of CART use for adults in the United States (US). In this retrospective study of patients who received CART therapy in the inpatient setting in 2018, we demonstrate that patients of minority racial/ethnic groups (especially Blacks) were underrepresented in CART use for the treatment of non‐Hodgkin's lymphoma, acute lymphoblastic leukemia, and multiple myeloma. We also demonstrate that a majority of CART therapies were performed in teaching hospitals located in urban areas, and that most CART therapy recipients were urban dwellers. Inhospital mortality following CART therapy was low, and we found no association between race or ethnicity and CART acute outcomes (mortality, toxicity, and resource use).

Non‐Hispanic Whites constituted most of the CART recipients in our study, consistent with reports from the Center for International Blood and Marrow Transplant Research (CIBMTR). In the CIBMTR analysis of all CART infusions performed in the US from 2016 to 2019 (2058 patients), 81% were Caucasians, while Asians and African Americans were 4% and 5%, respectively.^28^ An updated report from the CIBMTR continues to show a similar pattern for CART recipients from 2016 to 2021 (4094 patients, 78% non‐Hispanic Caucasians).^32^ Put together, these findings indicate that people of minority racial or ethnic origins are poorly represented in CART receipt in the United States, and based on the aforementioned CIBMTR reports, the trend has not significantly improved since 2016.

Racial disparity in CART receipt in our study affected Blacks more disproportionately compared to other minority racial groups. When the findings of our research are juxtaposed with the US census demographic data^33^ and prevalence and incidence data **(**Tables 4 and 5 **)**, receipt of CART therapies by Blacks is markedly low compared to their proportion of the US racial makeup and disease burden. While Blacks constituted 13.4% of the US population in 2019, only 4.2% of the total CART recipients in our study were reported to be Blacks. And, while multiple myeloma is 2–3 fold more common in African Americans compared to Caucasians,^34^, ^35^, ^36^ Blacks constituted only 15.8% of CART recipients for multiple myeloma in our study (compared to 71.1% Whites). A similar pattern was observed by the Pediatric Real World CAR Consortium, which reported that in a cohort of 200 recipients of tisagenlecleucel for relapsed and refractory ALL, only 5.5% were Blacks.^29^ Not only were Blacks and Asians poorly represented in the Pediatric Real World CAR Consortium study, but also Blacks were disproportionately represented among patients who were not infused with the CART product (36.4% of Blacks were not infused compared to 5.8% of patients of other races).

**TABLE 4:** Incidence rates, by race/ethnicity, 2013–2017, SEER 21 (rates per 100,000 population)

	NHL	ALL	MM
White (non‐hispanic)	21	1.6	6.3
Black	14.7	1.0	13.8
Hispanic	18.1	2.5	6.8
Asian/Pacific Islander	13.5	1.5	3.9
American Indian/Alaskan Native	10.9	1.6	5.7

Source: *Facts 2020–2021* and *Cancer Facts & Figures 2021*, American Cancer Society, 2021 and Surveillance, Epidemiology, and End Results Program.

NHL, Non‐Hodgkin's lymphoma; ALL, acute lymphoblastic leukemia; MM, multiple myeloma; SEER, Surveillance, Epidemiology, and End Results.

**TABLE 5:** Approximate United States prevalence of blood cancers, by race/ethnicity, as of January 1, 2017

	NHL	ALL	MM
White (non‐hispanic)	541,634	42,981	93,963
Black	54,040	5238	28,309
Hispanic	54,782	19,802	11,018
Asian/Pacific Islander	21,124	3465	3843
American Indian/Alaskan Native	1742	576	605

Source: *Facts 2020–2021* and *Cancer Facts & Figures 2021*, American Cancer Society*,* 2021 and Surveillance, Epidemiology, and End Results Program.

NHL, Non‐Hodgkin's lymphoma; ALL, acute lymphoblastic leukemia; MM, multiple myeloma.

While we found no differences in CART use patterns for the geographical regions of the US, we observed from our data that CART therapy was predominantly performed in teaching hospitals located in urban centers. This pattern is not surprising since CART infusions and subsequent care require subspecialty expertise and multidisciplinary care that are mostly available in large cancer centers. Subsequently, patients who live far from these teaching hospitals will likely experience poor access to CART treatments. In a retrospective evaluation of adult CART recipients from 2018 to 2020, Ahmed et al. found a relationship between patients' distance from the CART center, household income, and likelihood to undergo CART. In their analysis, the authors noted that patients who live greater than 60 miles from a CART center were less likely to undergo CART, especially if they lived in neighborhoods with a median income of <$40,000. The authors concluded that patients' residence location relative to the CART center (and their household income) influences CART access.^37^

The US Census Bureau estimates that about 19% of the US population lives in rural areas.^38^ But our study revealed that across disease types, only 14.7% of CART recipients were residents in rural areas. For multiple myeloma, in particular, CART recipients were almost exclusively residents of metropolitan counties (areas with population ≥50,000). The lower representation of rural residents in CART may be due to lower access to care, higher travel distance, and higher health‐related financial burdens compared to urban dwelling residents. Since all CART therapies for multiple myeloma in 2018 were given within the context of clinical trials, the extremely low representation of rural dwellers in receipt of CART for multiple myeloma calls for improved access of rural dwellers to clinical trials. With the current shortages of physicians, oncologists, and specialty cancer centers in rural America,^39^, ^40^ it is likely that rural dwellers may continue to experience a lack of access to CART therapies in the near future.

Even though racial and rural–urban disparities were noted in CART patterns of use, we found no association between race or residence and outcomes of CART treatment hospitalization. Inpatient mortality was relatively low, and acute CART toxicities were similar to rates reported in current literature. Even though persons of minority origin had a higher comorbidity burden compared to Whites, both groups had similar rates of acute CART complications and mortality. Resource utilization was also not significantly different between the two groups. Some studies have shown rural–urban disparities in health and all‐cause mortality,^41^, ^42^, ^43^ but we found no association between residence in the metropolitan areas and CART treatment outcomes. Compared to other urbanization areas, residents in the suburbs did not predict CART treatment outcomes.

Our study is limited by its retrospective nature. Since the NIS database lacks imaging and laboratory details, we relied on ICD‐10 codes to define acute CART complications. As such, our estimates of some acute CART toxicities may be lower than expected. Secondly, ICD‐10 codes for cytokine release syndrome did not exist in 2018, and we could not provide estimates of cytokine release syndrome from our data. Finally, our study evaluated CART therapies administered in the inpatient setting, but we have no reason to believe that our results would be significantly different if outpatient CART therapies were included in our analysis. Despite these few limitations, our analysis sets the stage for larger and prospective studies that would critically evaluate disparities in CART therapy use in the US.

In conclusion, this retrospective analysis of a large national inpatient database evaluated the patterns of CART therapy use across sociodemographic groups in the US and found that adults of minority racial origin were underrepresented in CART use. Racial disparity in CART use was more pronounced for Blacks. Americans living outside metropolitan areas were less likely to receive CART therapy. The extremely low inclusion of nonmetropolitan residents in CART receipt for multiple myeloma emphasizes the need to make CART therapies and clinical trials available beyond urban teaching hospitals. Our analysis revealed equivalent hospitalization outcomes irrespective of patients’ race or residence.

## FUNDING INFORMATION

This research did not receive any specific grant from funding agencies in the public, commercial or not‐for‐profit sectors.

## CONFLICT OF INTEREST

The authors declare no competing financial interests.

## AUTHOR CONTRIBUTIONS

Josephine Emole: Conceptualization, Methodology, Formal analysis, Writing ‐ original draft preparation, Writing ‐ review and editing.

Odunayo Lawal: Conceptualization, Writing ‐ review and editing.

Oleksandra Lupak: Writing ‐ review and editing.

Ajoy Dias: Writing ‐ review and editing.

Leyla Shune: Writing ‐ review and editing.

Korede Yusuf: Methodology, Writing ‐ original draft preparation, Writing ‐ review and editing, Supervision.

## Supporting information


Table A

## Data Availability

The data that support the findings of this study are available from The Healthcare Cost and Utilization Project, Agency for Healthcare Research and Quality. Restrictions apply to the availability of these data, which were used under a Data Use Agreement for this study. Data are available from https://www.hcup‐us.ahrq.gov/databases.jsp with the permission of the Agency for Healthcare Research and Quality.
