# Supplementary material for: Demographic differences among patients treated with chimeric antigen receptor T‐cell therapy in the United States
Source: Cancer Med. 2022 May 8;11(23):4440–8. doi: 10.1002/cam4.4797 (PMC9741965; doi:10.1002/cam4.4797)
Supplement: Supplementary file 1 — Table A [file CAM4-11-4440-s001.docx]

**Table A.** **Proportion of missing variables in study cohort**

| Variable | Categories | % Missing |
| --- | --- | --- |
| Age | Continuous variable (years) | 0 |
| Race | White/Black/Hispanic/Asian or Pacific Islander/Native American/Others | 5.5 |
| Gender | Female / Male | 0 |
| Payer (Insurance) | Medicare/Medicaid/Private/Self-Pay | 0.39 |
| Median Income | Continuous variable (US dollars) | 3.94 |
| Total Charges | Continuous variable (US dollars) | 0.39 |
| Hospital Teaching Status | Rural/Urban non-teaching/Urban teaching | 0 |
| Hospital Region | Northeast/Midwest/South/West | 0 |
| Hospital Location | New England/Middle Atlantic/East North Central/West North Central/South Atlantic/East South Central/West South Central/Mountain/Pacific | 0 |
| Length of Stay | Continuous variable (days) | 0 |
| NCHS Urban-Rural Code | Six-category codes 1/2/3/4/5/6 | 3.54 |

NCHS: National Center for Health Statistics
